# Optimization of the Preparation Process and Ameliorative Efficacy in Osteoporotic Rats of Peptide–Calcium Chelates from Skipjack Tuna (*Katsuwonus pelamis*) Meat

**DOI:** 10.3390/foods13172778

**Published:** 2024-08-30

**Authors:** Wan-Zhen Yan, Jiao Wang, Yu-Mei Wang, Yu-Hui Zeng, Chang-Feng Chi, Bin Wang

**Affiliations:** 1 Zhejiang Provincial Engineering Technology Research Center of Marine Biomedical Products, School of Food and Pharmacy, Zhejiang Ocean University, Zhoushan 316022, China; wanne_y@163.com (W.-Z.Y.); wangjiao1231234@163.com (J.W.); wangyumei731@163.com (Y.-M.W.); 2 National and Provincial Joint Laboratory of Exploration and Utilization of Marine Aquatic Genetic Resources, National Engineering Research Center of Marine Facilities Aquaculture, School of Marine Science and Technology, Zhejiang Ocean University, Zhoushan 316022, China; zengyuhui@zjou.edu.cn

**Keywords:** skipjack tuna (*Katsuwonus pelamis*), meat, peptide–calcium chelates, osteoporosis

## Abstract

This study aimed to establish the preparation process of peptide–calcium chelates (TMP-Ca) using skipjack tuna meat and investigate the function and mechanism of TMP-Ca in an osteoporosis model of rats. The results indicated that trypsin is more suitable for preparing the Ca-chelating hydrolysates of tuna meat, and the optimal hydrolysis conditions were derived as follows: digestion time 4 h, material–liquid ratio 1:10, and enzyme dose 3%. The conditions for chelating Ca with tuna meat hydrolysate were optimized to be chelation time 50 min, temperature 50 °C, pH 8.0, and a peptide–Ca ratio 1:10. The prepared hydrolysate was subjected to ultrafiltration, and the fraction (TMP) (MW <1 kDa) showed the highest Ca chelation rate (51.27 ± 1.42%) and was made into the peptide–Ca chelates (TMP-Ca). In osteoporotic rats, TMP-Ca significantly improved the decrease in ovarian indexes caused by retinoic acid. It also elevated serum Ca, phosphorus, and bone turnover indexes, increased the number of bone trabeculae, and improved bone microstructure. In addition, we confirmed that TMP-Ca could regulate the OPG/TRAF6 pathway to reduce osteoclast differentiation, inhibit bone resorption, and promote bone formation. Therefore, TMP-Ca could significantly ameliorate osteoporosis, and this study provides a functional component for the preparation of healthcare products using skipjack tuna meat to treat osteoporosis.

## 1. Introduction

Bioactive peptides, derived from specific small-molecule amino acid sequences in nature, usually consist of 3 to 20 amino acids, whose unique amino acid composition, sequence arrangement, and molecular weight (MW) characteristics enable them to exert significant physicochemical effects in the normal metabolic processes of organisms [1,2]. A wide range of complex physiological functions in organisms are essentially based on the interactions between amino acids [3,4,5]. Bioactive peptides extracted from abundant and diverse food resources not only retain the superiority of their natural sources, but also increasingly become the focus of drug discovery and development due to their unique advantages in enhanced permeability, low toxicity, and rapid in vivo clearance. These advantages have led to the widespread application of therapeutic bioactive peptides in a variety of industries, such as pharmaceuticals, food, and daily chemicals, showing great potential for development [6,7,8]. Recently, there has been growing interest in the calcium (Ca) chelates of food-borne peptides due to their enhanced stability, absorption, and bioavailability [9,10,11]. These chelates are formed by combining protein hydrolysates with calcium ions, providing both Ca and peptides, making them a promising new type of Ca supplement [11]. Therefore, peptide–Ca chelates have been produced successfully from animal and plant proteins, such as monkey mushrooms [12], peanuts [13], glycated walnut meal [14], rice [15], chicken blood [16], and beef bones [17]. Meanwhile, much progress has been made in peptide–Ca chelates from aquatic organisms, such as novel peptides with Ca-chelating activity prepared using jellyfish [18], Antarctic krill [19], octopus by-products [20], oysters [21], tuna bone [22], and tilapia bone [23]. Studies have also shown that peptide–Ca chelates can significantly increase rats’ Ca absorption rate, Ca retention rate, and femoral Ca content [24].

Population aging is a global trend, and attention to age-related diseases, such as osteoporosis (OP), is increasing [25]. OP and its complications, such as osteoporotic fractures, pain, and limited mobility, pose a significant threat to patients’ physical and mental health [26]. Although various anti-OP products are available in the clinic, their long-term application is limited due to their high cost and adverse effects [27]. Therefore, it is essential to develop affordable and effective anti-OP products without any toxic side effects [28]. A study by Liu revealed a significant increase in femoral bone index, serum Ca level, serum osteocalcin level, and Ca content of the femur when rats were fed a high dose of casein phosphopeptide Ca chelate for seven weeks [29]. Su et al. extracted specific peptides from *Muraenesox cinereus* bones and chelated them with Ca, and found that this peptide–Ca chelates significantly enhanced Ca transport efficiency in Caco-2 cells and promoted Ca deposition in MC3T3-E1 cells. In the zebrafish OP model, peptide–Ca chelates increased bone mineral density and raised expression levels of ALP, OCN, OPG, and TGF-β while decreasing the expression of TRAP [25]. Wang et al. successfully purified a novel Ca-binding peptide of FPPDVA from the peanut protein hydrolysate, and found that FPPDVA-Ca chelate could effectively uptake and transport Ca through Cav1.3 and TRPV6 calcium channels in the rat intestinal bursa exocytosis experiments and the monolayer experiments in Caco-2 cells [13]. Li et al. successfully developed a supplement of deer sinew peptide–calcium chelates, which was found to promote the proliferation of MC3T3-E1 cells in vitro. In animal experiments, it was found that the product could regulate serum markers and improve bone microstructure changes caused by Ca deficiency. Moreover, the expression levels of calcium absorption genes (Trpv5, Trpv6, CaBP-d9k, and PMCA1b) in the kidney were up-regulated [30].

Tuna is a low-fat, low-calorie, and high-protein fish with high bioavailability value, making it a precious marine resource [31,32]. The tuna processing industry has seen continuous development, resulting in the popularity of products such as sashimi and canned tuna [33]. However, the sector often neglects the waste produced during processing, including heads, skins, bones, offal, and minced meat, leading to a waste of resources [34,35]. Research has shown that tuna meat peptides possess a range of biological activities, including anti-fatigue, anti-hypertensive, anti-aging, lipid-lowering, antioxidant, anti-inflammatory, and immunomodulatory effects [9,36]. However, there is no corresponding Ca-chelating structure and function study of peptides from tuna meat. Therefore, the objectives of this investigation were to establish the preparation process of peptide–Ca chelates (TMP-Ca) using skipjack tuna (*Katsuwonus pelamis*) meat and to explore the function and mechanism of TMP-Ca on the retinoic acid-induced osteoporosis model of rats. This study enhances the value of minced tuna meat. It also provides a scientific basis for the development of new functions and products against osteoporosis.

## 2. Materials and Methods

### 2.1. Materials, Reagents and Instruments

Skipjack tuna (*K. pelamis*) meat was supplied by Jiri Food Co., Ltd. (Ningbo, China). Trypsin, papain, neutral protease, and alkaline protease were purchased from Gibco Life Sciences (New York, NY, USA). The calcium carboxylic acid indicator was purchased from Sinopharm Co., Ltd. (Shanghai, China). Triethanolamine was purchased from Sigma Co., Ltd. (Shanghai, China). Retinoic acid and alendronate were purchased from Shanghai Yuanye Biotechnology Co., Ltd. (Shanghai, China). The calcium kits, phosphorus kits, alkaline phosphatase (ALP) kits, anti-tartaric acid phosphatase (TRAP) kits, osteoprotegerin (BGP) kits, and the ECL Luminous color-developing solution were purchased from Nanjing Jiancheng Biological Co., Ltd. (Nanjing, Jiangsu, China). GAPDH, OPG, and TRAF6 were purchased from Wuhan Sanying Biotechnology Co., Ltd. (Wuhan, Hubei, China).

LGJ-10D freeze dryer was purchased from Beijing Sihuan Scientific Instrument Factory (Beijing, China). TGL-16G high-speed centrifuge was purchased from Shanghai Anting Scientific Instrument Factory (Shanghai, China). BSA124S Electronic Balance was purchased from Sartorius Instrument Co., Ltd. (Beijing, China). The WTM-1812 membrane separation system was purchased from Hangzhou Woten Membrane Engineering Co., Ltd. (Hangzhou, China). A −20 °C low-temperature refrigerator was purchased from Midea Refrigerator Co., Ltd. (Shanghai, China). A −80 °C thermal U/L refrigerator and Multisjan F microplate reader were purchased from Thermo Fisher Technology Co., Ltd. (Beijing, China). An HH-W420 thermostatic water tank was purchased from Changzhou Jintan Hengfeng Instrument Manufacturing Co., Ltd. (Changzhou, Jiangsu, China). TI-S inverted fluorescence biomicroscopy was purchased from Nikon Corporation (Tokyo, Japan). A DF-101S heating type constant temperature heating magnetic stirrer was purchased from Zhengzhou Ketai Experimental Equipment Co. Ltd. (Zhengzhou, Henan, China). Mini-Protean Tetra vertical electrophoresis was purchased from Bio-Rad Laboratories (Shanghai, China). The FC3 Gel Imaging System was purchased from ProteinSimple Company (San Jose, CA, USA).

### 2.2. Preparation of Skipjack Tuna Meat Peptide–Ca Chelates (TMP-Ca)

#### 2.2.1. Pre-Treatment of Skipjack Tuna Meat

Skipjack tuna meat was thawed and stripped of impurities, such as fish bones and scales. The remaining fish meat was washed with running water and selected as experimental material. The fish meat was then dried in a drying oven (37 °C) and ground into powder. After that, the tuna meat powder was degreased by adding anhydrous ethanol at a 1:4 (*w*/*v*) ratio for 10 h. After degreasing, the solution was removed with a filter, and the residues were dried and stored at −20 °C.

#### 2.2.2. Screening of Optimal Proteases

A total of 5 g of tuna defatted powder was separately hydrolyzed by trypsin, papain, neutral protease, and alkaline protease under optimal conditions. The reaction was carried out for 4 h at a material to liquid ratio of 1:10 and 2% enzyme dose [37]. After the enzymatic reaction, the pH of the enzymatic solution was adjusted to 7 and the enzyme was inactivated at 95 °C for 10 min. Finally, the enzymatic solution was centrifuged at 8000 rpm to collect the supernatant, which was then lyophilized and stored at −20 °C for later use. The Ca chelation rates of skipjack tuna meat hydrolysates were determined, and hydrolysate prepared by trypsin had the highest Ca chelation rate.

#### 2.2.3. Single-Factor Experiment for Optimizing Hydrolysis Conditions of Trypsin

Based on the above experimental results, trypsin was selected as the experimental enzyme, and the hydrolysis conditions of trypsin including enzyme dose (1%, 2%, 3%, 4%, and 5%), hydrolysis time (1, 2, 3, 4, and 5 h), and tuna meat–liquid ratios (1:5, 1:10, 1:15, 1:20, and 1:25) were optimized using single factor experiment [19].

#### 2.2.4. Determination of Ca Chelation Rate

EDTA titration was used to assess the total quantity of Ca in the chelating (C_1_) solution as well as the Ca content in the supernatant (C_2_) after centrifugation [22]. The determination of the chelating capability was conducted in the following manner:

(1) $$\mathrm{Chelation}\;\mathrm{rate}\;(\%)=(C_{1}-C_{2})/C_{1}\times 100\;\%$$

#### 2.2.5. One-Way Experimental Design of Chelation Reaction

Tuna meat hydrolysate solution with a concentration of 1% was created by dissolving 0.3 g of hydrolysate power in 30 mL of deionized water, followed by the addition of anhydrous CaCl_2_ at the designed hydrolysate–Ca ratio. After that, using the Ca chelation rate as an index, the chelation process was optimized by varying the chelation time (30, 40, 50, 60, and 70 min), chelation temperature (30, 40, 50, 60, and 70 °C), chelation pH (6.0, 7.0, 8.0,9.0, and 10.0), and hydrolysate–Ca ratio (1:5, 1:10, 1:15, 1:20, and 1:25). Following the completion of the chelation reaction, nine times the volume of anhydrous ethanol was added in the solution and the mixture was deposited in a 4 °C refrigerator overnight. Following this, the mixture was centrifuged at 8000 rpm for 10 min, and the resulting residue was freeze-dried and measured its Ca chelation rate according to the described method in Section 2.2.4.

#### 2.2.6. Ultrafiltration of Tuna Meat Hydrolysate

The tuna meat hydrolysate, which was treated with trypsin, underwent ultrafiltration using membranes with molecular weight (MW) cutoffs of 1, 3, 5, and 10 kDa. Five peptide components were obtained: those with a MW less than 1 kDa, between 1 and 3 kDa, between 3 and 5 kDa, between 5 and 10 kDa, and greater than 10 kDa. The Ca chelation rates of these components were determined. The fraction with a MW less than 1 kDa exhibited the highest Ca chelation rate and was designated as TMP. The resulting Ca chelate of TMP was named TMP-Ca chelates.

### 2.3. Ameliorative Function of TMP-Ca Chelates on Retinoic Acid-Induced Osteoporosis Model in Rats

#### 2.3.1. Animals and Treatments

The rat model of osteoporosis induced by retinoic acid was established following the procedure outlined by Oršolić [38] with minor adjustments. The blank group received a daily gavage of 0.9% saline, while the other five groups were administered 75 mg/kg retinoic acid for two consecutive weeks. During the experimental period, the blank and model groups continued to be gavaged with saline, the positive control group was gavaged with 5 mg/kg alendronate, and the low-, medium-, and high-dose groups of TMP-Ca chelates were gavaged with 400, 600, and 800 mg/kg TMP-Ca chelates, respectively. Body weights were recorded weekly and measured after 12 h of fasting following gavage. Subsequently, blood samples were collected from the rats under anesthesia, centrifuged, and serum frozen. The viscera were removed and weighed, and the femur and tibia were rinsed with saline to remove excess tissue and then fixed in a 4% paraformaldehyde solution for subsequent analysis.

#### 2.3.2. Determination of Organ Indices in Rats

The organ index of the rats was determined using the following formula after the body weights and the weights of the heart, liver, spleen, kidney, and ovary of each group of the rats were measured [39].

(2) Organ index (%)=Weight of the organs (g) / Weight (g)×100 %

#### 2.3.3. Determination of Serum Biochemical Parameters

Serum Ca content in the rats was determined according to the kit instruction based on the principle of the Methyl Thymol Blue (MTB) method. The content of serum phosphorus (P) in the rats was determined by the phosphomolybdic acid method according to the kit instructions. The rats in each group had their serum ALP, TRAP, and BGP levels determined according to the kit instructions.

#### 2.3.4. Determination of Bone Parameters

After removing the excess tissue from the femur and tibia of the rats, the length and diameter of the femur and tibia of the rats were measured using electronic vernier calipers, and the data were recorded. After the measurement, the bone tissue was stored in 4% paraformaldehyde.

The femur and tibia of the rats were carefully removed from the 4% paraformaldehyde. The residual liquid on the surface of the bone tissue was carefully dried with absorbent paper, then the bone tissue was weighed, and the data were recorded as wet bone weight. After being dried for two hours at 110 °C, the tibia and femur were weighed, and the results were reported as dry bone weight.

The femoral tissue section specimens were prepared using conventional paraffin sections, and the histopathologic changes in the femur were analyzed using H&E staining, and Mason-staining techniques [40].

### 2.4. Determination of the Protein Expression of Osteoporosis-Related Pathways

The rat tibias were frozen in liquid nitrogen and then ground under ultra-low temperature conditions (−80 °C~−100 °C). Protein lysates were prepared with radioimmunoprecipitation assay (RIPA) buffer containing a combination of protease and phosphate inhibitors. Each 10 μg protein sample was electrophoresed in a 10% SDS-PAGE gel and transferred to a PVDF membrane. The membrane was conjugated with a primary antibody overnight at 4 °C. The following day, it was exposed to secondary anti-mouse or anti-rabbit antibodies for one hour [41].

### 2.5. Data Statistics

All the data were expressed as mean ± SD (*n* = 8) and statistically analyzed using the SPSS software (version 27.0). A *t*-test for independent samples was used to analyze the significance of differences, with *p* < 0.05 being considered statistically significant.

## 3. Results

### 3.1. Preparation of TMP-Ca Chelates

#### 3.1.1. Optimization of the Enzymatic Hydrolysis Process

**Figure 1:**
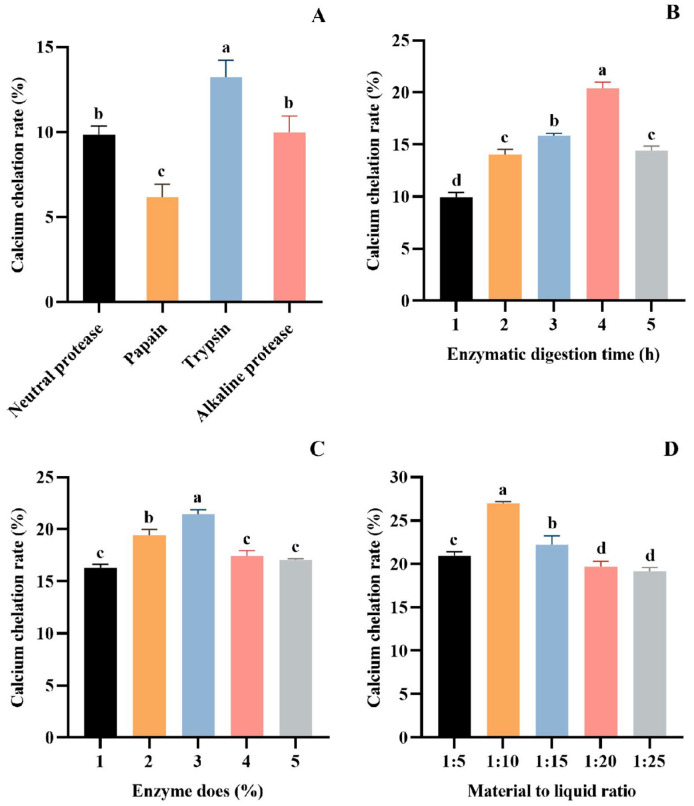
Effect of different proteases (**A**), hydrolysis time (**B**), enzyme dose (**C**), and material–liquid ratio (**D**) on Ca chelation rate of tuna meat hydrolysates. All values are means ± SD (*n* = 3). ^a–d^ No significant difference between the same letters (*p* > 0.05).

The protein hydrolysates obtained under different treatment conditions significantly affected the chelating ability of Ca [42,43]. Currently, the most effective method for obtaining bioactive peptides is enzymatic hydrolysis [44,45]. It has been shown that proper enzymatic hydrolysis promotes the exposure of hydrophilic groups and enhances Ca coordination, which can significantly increase the rate of Ca chelation [46]. In order to obtain the protein hydrolysate with the optimal chelation of calcium ions, the proteases were screened by Ca chelation rate as the index. The results showed that trypsin is the most suitable for preparing hydrolysate with the best Ca chelating effect (13.23 ± 0.99%) (*p* > 0.05), followed by alkaline protease (9.96 ± 0.98%), neutral protease (9.85 ± 0.50%), and papain (6.16 ± 0.77%) (Figure 1A). Therefore, trypsin was chosen as the experimental enzyme.

The parameters of trypsin on the Ca chelation rate of tuna meat hydrolysate were investigated in a single-factor experiment. As seen from Figure 1B, the Ca chelation of the prepared hydrolysate was the first to increase and decrease with the extension of the enzymatic digestion time. The Ca chelation rate of the hydrolysate prepared for 4 h reached 20.41 ± 0.57% (*p* > 0.05), which was significantly higher than other groups. Figure 1C depicts the influence of enzyme dose (1%, 2%, 3%, 4%, and 5%) on the Ca chelation rate of tuna meat hydrolysate. The maximum Ca chelation rate was achieved at a 3% enzyme dose, with a chelation rate of 21.46 ± 0.42% (*p* > 0.05). In addition, the increase in the Ca chelation rate of the hydrolysate tended to level off at the enzyme dose level of more than 3%. Figure 1D presents the effect of material–liquid ratios (1:5, 1:10, 1:15, 1:20, and 1:25) on the Ca chelation rate of tuna meat hydrolysate. At a material–liquid ratio of 1:10, the produced hydrolysate showed the maximum Ca chelation (26.99 ± 0.19%) (*p* > 0.05). Then, the optimized conditions of trypsin for hydrolyzing defatted tuna meat were a hydrolysis time of 4 h, enzyme dose of 3%, and material–liquid ratio of 1:10.

#### 3.1.2. Optimization of Chelating Conditions of Protease Hydrolysate and Ca

**Figure 2:**
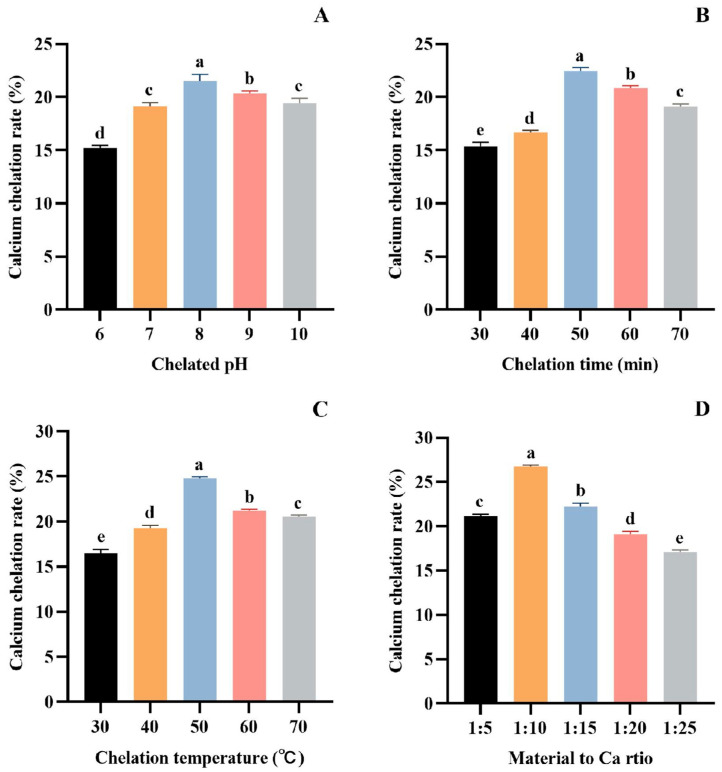
Effect of chelation pH (**A**), time (**B**), temperature (**C**), and material to Ca ratio (**D**) on the Ca chelation rate of the tuna meat hydrolysates. All the values are means ± SD (*n* = 3). ^a–e^ No significant difference between the same letters (*p* > 0.05).

As seen in Figure 2A, the Ca chelation rate of tuna meat hydrolysate gradually increased in the range of pH 6.0~8.0, reached the maximum value of 21.53 ± 0.59% (*p* > 0.05) at pH 8.0, and decreased in the range of pH 8.0~10.0. At a low pH, H^+^ in the reaction solution will compete with Ca^2+^ for electron donors, thus affecting the peptide’s complexation with calcium ions. With the increase in pH, H^+^ in aqueous solution will be diluted, and the complexation between peptide and Ca^2+^ can be enhanced [47,48]. However, under alkaline conditions, OH^−^ will preferentially bind to the electron donor group of Ca^2+^ to form a calcium hydroxide precipitate. Therefore, peptides are more easily combined with calcium salts to form chelates at pH 8.0.

As seen in Figure 2B, the Ca chelation rate of tuna meat hydrolysate increased gradually with the reaction time ranging between 30 and 50 min, and reached the highest value (22.46 ± 0.32%) (*p* > 0.05) when the chelation time was 50 min. This phenomenon is because the formation of the peptide–Ca complex is a dynamic adsorption process. At 50 min, the adsorption and dissolution processes reached a dynamic equilibrium. Therefore, the optimum time for the chelation of tuna peptides with Ca is 50 min.

Figure 2C demonstrated that the Ca chelation rate of tuna meat hydrolysate was as high as 24.79 ± 0.17% (*p* > 0.05) at 50 °C, indicating that the Ca chelating reaction was most active at this temperature. Above 50 °C, the structure of the peptide–Ca complex is easily destroyed, which affects the reaction of Ca^2+^ with peptides, and thus reduces the rate of Ca chelation. Therefore, the optimum temperature for the Ca chelation rate of tuna meat hydrolysate is 50 °C.

Figure 2D depicts that the Ca chelation rate of tuna meat hydrolysate reached a maximum value (26.76 ± 0.14%) (*p* > 0.05) when the ratio of material–Ca was 1:10. However, the chelation rate showed an obvious downward trend with the increase in Ca^2+^ when the material–Ca ratio was more than 1:10, indicating that the peptide ligand was insufficient. Therefore, the material–Ca ratio of 1:10 was selected in the subsequent experiments.

#### 3.1.3. Ultrafiltration Fractionation of Tuna Meat Hydrolysate

**Figure 3:**
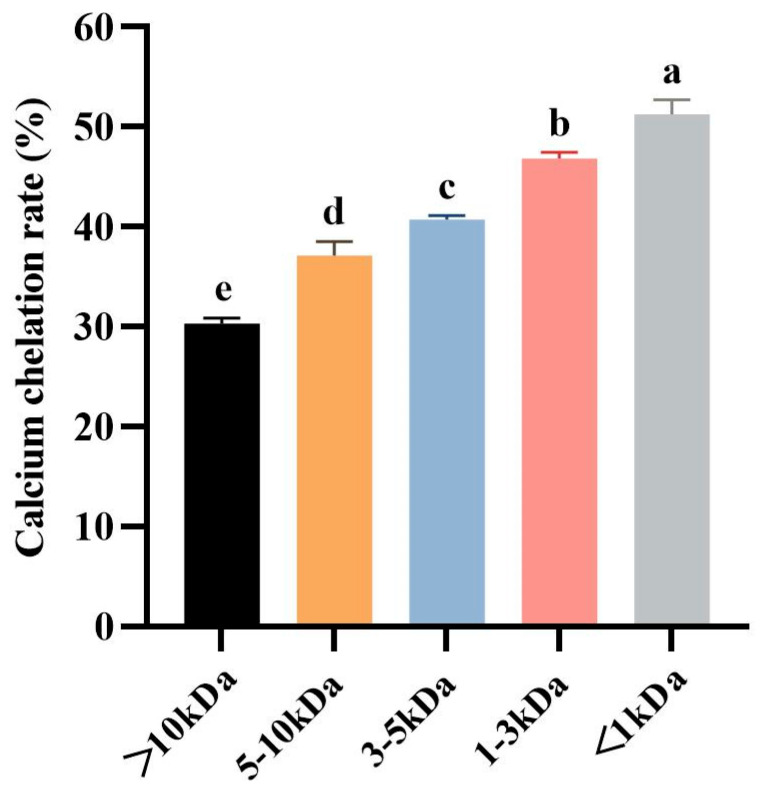
The Ca chelation rates of the five peptide fractions from the tuna meat hydrolysate. All the values are means ± SD (*n* = 3). ^a–e^ No significant difference between the same letters (*p* > 0.05).

Molecular weight (MW) is the most important factor to consider for the separation of fractions with desired functional or biological properties from protein hydrolysates [49]. Ultrafiltration is a technique for separating molecules in solution based on molecular size, and it is widely used to concentrate target fractions from protein hydrolysates on a MWCO membrane [50]. Therefore, the defatted tuna meat powder was fully digested by trypsin under the optimized conditions, and the prepared hydrolysate was fractionated by ultrafiltration to obtain five fractions with molecular weights less than 1 kDa, between 1 and 3 kDa, between 3 and 5 kDa, between 5 and 10 kDa, and greater than 10 kDa, respectively. Five fractions were chelated with CaCl_2_, and then their Ca chelation rates were determined (Figure 3). The results showed that the Ca chelation rate of <1 kDa fraction was 51.27 ± 1.42% (*p* > 0.05), which was significantly higher than the other four fractions. This result indicated that small molecule peptides are more likely to bind with Ca^2+^ to generate peptide–Ca chelates. Therefore, the chelate of <1 kDa fraction and Ca^2+^ was named as TMP-Ca and chosen for the subsequent studies.

### 3.2. Ameliorative Effect of TMP-Ca Chelates on Retinoic Acid-Induced Osteoporosis in Rats

#### 3.2.1. Effect of TMP-Ca Chelates on Body Weight of Model Rats

**Figure 4:**
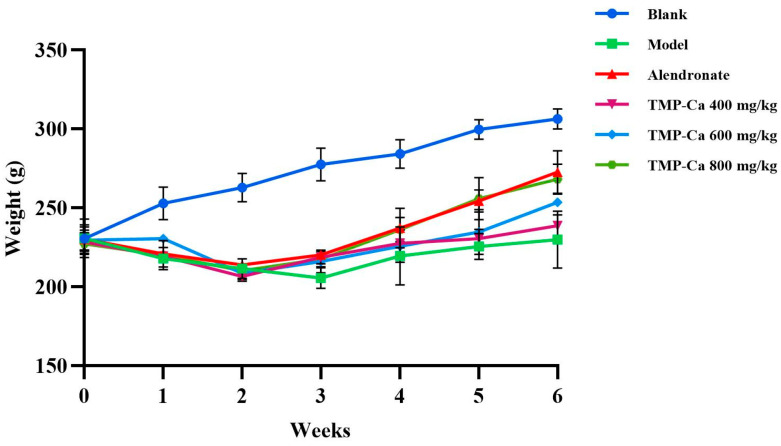
Effect of TMP-Ca chelates on body weight of model rats. Blank control: 0.9% saline; model: 0.9% saline + retinoic acid; positive control (alendronate): 5 mg alendronate/kg + retinoic acid; low dose of TMP-Ca: 400 mg/kg + retinoic acid; medium dose of TMP-Ca: 600 mg/kg + retinoic acid; high dose of TMP-Ca: 800 mg/kg + retinoic acid.

Figure 4 depicts the changes in rat body weight in each group. Following the initiation of retinoic acid administration, the body weight of the rats in each group started to exhibit distinct trends. After 2 weeks of gavage, all the rats treated with retinoic acid showed weight loss, slowed movement, slowed response, reduced water intake, and yellowed and loose body hair. Previous studies have shown that retinoic acid reduces the appetite of rats, which further leads to decreased rat food intake and weight loss, indicating that the retinoic acid-induced osteoporosis model of rats has been successfully established [38]. In addition, the body weights of the rats in the positive control group and TMP-Ca chelate groups increased progressively and were higher than the rate of body weight increase in the rats in the model group. After four weeks, when compared to the model group, the TMP-Ca chelate groups’ body weight increased considerably, and the body hair and other physical characteristics of the rats recovered significantly, indicating that the TMP-Ca chelates may be crucial in controlling the osteoporotic rats’ state of health.

#### 3.2.2. Analysis of Rat Organ Index

Through the determination of the main organ indexes of the rats (Table 1), we found that the retinoic acid treatment resulted in a significant increase in the heart, liver, spleen, and kidney indexes of the rats in each group, which was mainly attributed to the specific damage effect of retinoic acid on the liver. It is particularly noteworthy that the ovarian index of the rats in the model group was only 0.06 ± 0.32%, considerably less than what was found in the other groups. However, after the intervention of the TMP-Ca chelates, the ovarian index of the rats showed a recovery trend. In particular, the ovarian index of the high-dose group (0.16 ± 0.91%) was similar to that of the blank group (0.16 ± 0.23%).

Prior studies have confirmed that retinoic acid is a reproductive toxin and can destroy the gonad, leading to an imbalance of bone metabolism in the body, thereby inducing osteoporosis [51,52]. The decrease in ovarian capacity noted in the present experiment fits well with the descriptions in these reports. In summary, this study’s findings suggest that TMP-Ca chelates have a certain repair effect on ovarian damage in the retinoic acid-induced osteoporosis rats.

#### 3.2.3. Analysis of Serum Biochemical Parameters

**Figure 5:**
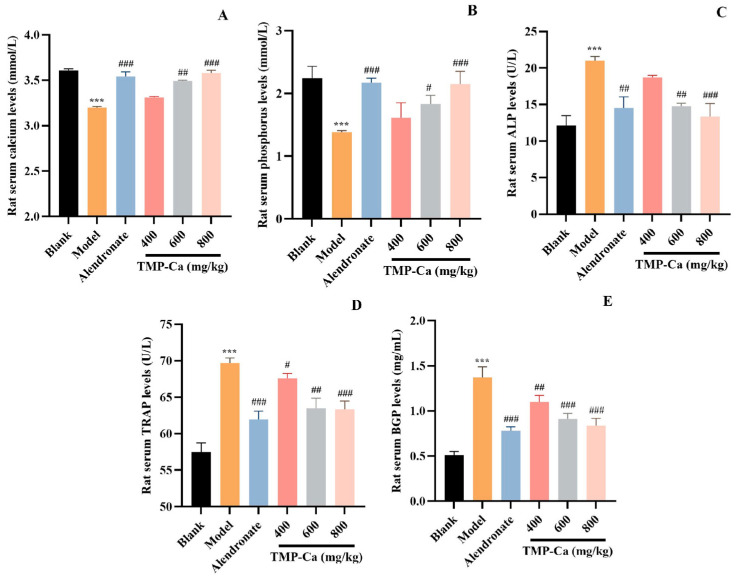
Effect of TMP-Ca chelates on rat blood biochemical indicators. (**A**) Serum calcium; (**B**) serum phosphorus; (**C**) alkaline phosphatase; (**D**) acid phosphatase; (**E**) osteocalcin. All values are means ± SD (*n* = 8). ^***^ *p* < 0.001 vs. blank group; ^#^ *p* < 0.05, ^##^ *p* < 0.01, and ^###^ *p* < 0.001 vs. model group.

The results of several serum biochemical markers related to bone metabolism are shown in Figure 5. The serum Ca level of the model group was 3.20 ± 0.01 mmol/L (*p* < 0.001), which was significantly lower than that (3.61 ± 0.02 mmol/L) of the blank group. However, the serum Ca level in the TMP-Ca chelate group was increased, and the serum Ca level in the high-dose group was significantly increased to 3.58 ± 0.03 mmol/L (*p* < 0.001). As shown in Figure 5B, the serum P level of the model group (1.38 ± 0.03 mmol/L) (*p* < 0.001) was lower than that of the blank group (2.24 ± 0.19 mmol/L). However, the decline caused by retinoic acid was reversed by TMP-Ca chelates, and the serum P level in the high-dose group of TMP-Ca chelates was significantly increased to 2.15 ± 0.21 mmol/L (*p* < 0.001). The changes in serum Ca and P levels indicated that the TMP-Ca chelates could promote Ca absorption and inhibit retinoic acid-induced Ca and P loss in rats.

As illustrated in Figure 5C, compared with the blank group, the serum ALP activity of the model group was significantly increased to 21.01 ± 0.60 U/L (*p* < 0.001). The serum ALP level of TMP-Ca chelate groups decreased significantly, and the serum ALP level of the low-dose group decreased to 18.72 ± 0.30 U/L (*p* < 0.001). The serum ALP level of the high-dose group went down to 13.34 ± 1.80 U/L (*p* < 0.001), indicating that the TMP-Ca chelates could significantly reduce serum ALP. As illustrated in Figure 5D, in contrast to the blank group, the serum TRAP activity of the model group was significantly increased to 69.69 ± 0.70 U/L (*p* < 0.001), but the TMP-Ca chelates reversed the retinoic acid-induced rising trend, and the medium and high dose of the TMP-Ca chelates significantly reduced the serum TRAP activity to 63.47 ± 1.39 U/L and 63.34 ± 1.12 U/L (*p* < 0.001), respectively. The results showed that the TMP-Ca chelates could effectively reduce bone turnover and restore calcium absorption to normal levels.

As seen in Figure 5E, the rats in the model group had serum BGP levels of 1.37 ± 0.20 mg/mL (*p* < 0.001), which was considerably higher than rats in the blank group (0.51 ± 0.04 mg/mL). However, the serum BGP levels in the medium-dose (0.91 ± 0.06 mg/mL) (*p* < 0.001) and the high-dose (0.84 ± 0.08 mg/mL) (*p* < 0.001) groups of the TMP-Ca chelates showed a significant downward trend in comparison to the model group. This finding implies that TMP-Ca chelates can successfully limit the rise in the osteoclast and osteoblast activity brought on by retinoic acid, restoring a balance between bone production and resorption and helping to lessen osteoporosis symptoms.

#### 3.2.4. Analysis of Bone Parameters

**Figure 6:**
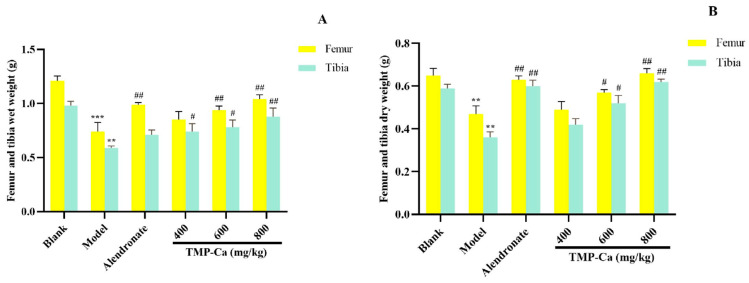
Effect of TMP-Ca chelates on wet weight (**A**) and dry weight (**B**) of femur and tibia in retinoic acid-induced rats. All values are means ± SD (*n* = 8). ^**^ *p* < 0.01 and ^***^ *p* < 0.001 vs. blank group; ^#^ *p* < 0.05, ^##^ *p* < 0.01, vs. model group.

As shown in Figure 6, the dry and wet weights of the femur and tibia in the model group (*p* < 0.001) were significantly lower than those in the blank group. However, after TMP-Ca chelate treatment, we found that the wet weight of the femur in the middle-dose and the high-dose groups of TMP-Ca chelates was 0.94 ± 0.03 g (*p* < 0.01) and 1.04 ± 0.04 g (*p* < 0.01), respectively, significantly higher than that in the model group. For the tibia, the wet weight of the middle-dose (0.78 ± 0.06 g) (*p* < 0.01) and high-dose (0.88 ± 0.07 g) (*p* < 0.01) groups of TMP-Ca chelates was also significantly higher than that of the model group (0.59 ± 0.01 g) (Figure 6A). In addition, Figure 6B showed that the femur dry weight of the TMP-Ca chelate high-dose group (0.66 ± 0.02 g) (*p* < 0.01) was significantly higher than that of the model group (0.47 ± 0.04 g). Similarly, the dry weight of the tibia in the middle- and high-dose groups of TMP-Ca chelates was 0.42 ± 0.02 g (*p* < 0.01) and 0.62 ± 0.01 g (*p* < 0.01), respectively, which was also significantly higher than that in the model group (0.36 ± 0.02 g). This result suggests that the intervention of the TMP-Ca chelates can effectively inhibit bone loss and have a clear positive effect on bone growth.

**Figure 7:**
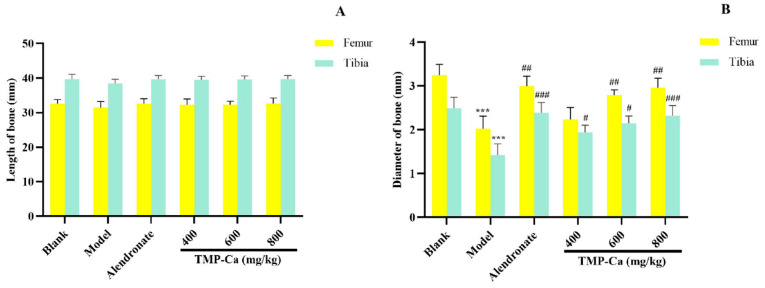
Effect of TMP-Ca chelates on the length (**A**) and diameter (**B**) of the femur and tibia in the retinoic acid-induced rats. All the values are means ± SD (*n* = 8). ^***^ *p* < 0.001 vs. blank group; ^#^ *p* < 0.05, ^##^ *p* < 0.01, and ^###^ *p* < 0.001 vs. model group.

As shown in Figure 7A, the femur and tibia lengths of the blank group, model group, and TMP-Ca chelate groups did not differ significantly from one another. However, Figure 7B indicated that the femur diameter of the rats treated with retinoic acid was significantly reduced to 2.02 ± 0.19 mm (*p* < 0.001) compared with the blank group (3.25 ± 0.12 mm), and the femur diameter of the middle- and high-dose groups of TMP-Ca chelates separately increased to 2.23 ± 0.13 mm (*p* < 0.01) and 2.96 ± 0.12 mm (*p* < 0.01).

Furthermore, the tibia diameter of the rats in the model group (1.42 ± 0.26 mm) (*p* < 0.001) was significantly smaller than that of the blank group (2.49 ± 0.25 mm), and the tibia diameter of the rats increased significantly to 2.32 ± 0.23 mm (*p* < 0.001) after the high-dose TMP-Ca chelate treatment. The results showed that intervention with the TMP-Ca chelates could effectively ameliorate bone damage and bone mass loss induced by retinoic acid in osteoporotic rats, and had a favorable impact on bone formation.

**Figure 8:**
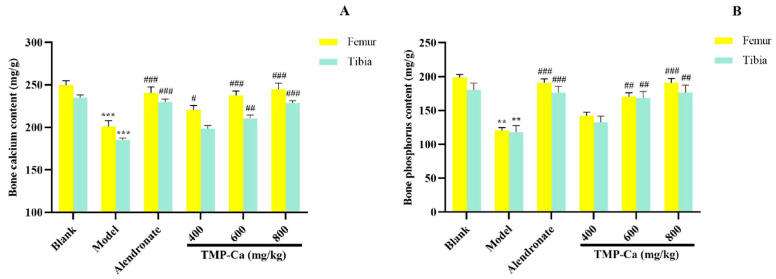
Effect of TMP-Ca chelates on the bone calcium (**A**) and phosphorus (**B**) contents of the femur and tibia in the retinoic acid-induced rats. All the values are means ± SD (*n* = 8). ^**^ *p* < 0.01 and ^***^ *p* < 0.001 vs. blank group; ^#^ *p* < 0.05, ^##^ *p* < 0.01, and ^###^ *p* < 0.001 vs. model group.

Figure 8 depicts the influence of the TMP-Ca chelates on the Ca and *p* contents of the femur and tibia in the retinoic acid-induced rats. Figure 8A indicated that the femur Ca content of the model group was 201.09 ± 6.75 mg/g (*p* < 0.001), which was substantially less than the blank group (249.95 ± 5.09 mg/g). Moreover, the femur Ca content of the high-dose TMP-Ca chelate group was significantly increased to 245.08 ± 6.92 mg/g (*p* < 0.001). For the tibia, the Ca content of the model group (186.09 ± 2.30 mg/g) (*p* < 0.001) was notably lower than that in the blank group (235.71 ± 2.96 mg/g), but it was significantly increased to 229.92 ± 2.76 mg/g (*p* < 0.001) after the high-dose TMP-Ca chelate treatment.

As seen in Figure 8B, the femoral *p* content of the model group (120.62 ± 4.35 mg/g) (*p* < 0.001) was notably lower than that of the blank group (198.83 ± 4.34 mg/g), but the high-dose TMP-Ca chelate treatment could significantly increase the *p* content to 190.99 ± 6.36 mg/g (*p* < 0.001). For the tibia, the *p* content of the model group (118.06 ± 9.67 mg/g) (*p* < 0.001) was significantly lower than that of the blank group (180.59 ± 9.84 mg/g), and the *p* content significantly increased to 176.62 ± 10.89 mg/g (*p* < 0.001) after the high-dose TMP-Ca chelate treatment.

The decrease in bone Ca and *p* in the model group confirmed that the metabolism of Ca and *p* in rats was in a negative equilibrium state, and the loss of Ca and *p* was serious, leading to bone loss and osteoporosis (Figure 8). The contents of the bone Ca and *p* in the TMP-Ca chelate groups were notably higher than those in the model group. These data proved that the TMP-Ca chelates could effectively alleviate retinoic acid-induced bone mineral loss, thereby improving bone quality.

#### 3.2.5. Histomorphological Effect of TMP-Ca Chelates on Rat Femur

##### Masson Staining Analysis

**Figure 9:**
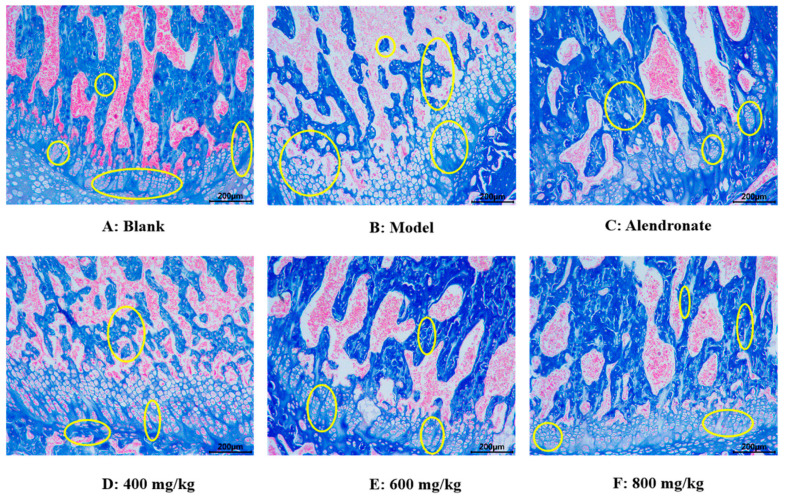
Effect of TMP-Ca chelates on histomorphology of rat femoral head using Masson staining method. Alendronate (5 mg/kg) was used as a positive control. Yellow circles indicate bone trabeculae and epiphyses.

Using the Masson staining method, the effect of TMP-Ca chelates on histomorphology longitudinal sections of the rat femur head was observed (Figure 9). In the blank group, collagen in the bone trabeculae of the rat femur appeared blue, the bone trabeculae were closely distributed, the bone was dense, the intracavernous hematopoietic cells were closely distributed, and then the epiphyseal of the femur shows a normal shape (Figure 9A). However, in the model group, the number of voids in the bone trabeculae of the rats given retinoic acid was higher, and the deformation of epiphysis appeared, showing significant symptoms of osteoporosis (Figure 9B). Compared with the model group, the number of cavities in TMP-Ca-treated rat femur decreased, especially the destruction of bone trabeculae was well controlled, indicating that TMP-Ca can inhibit the osteoporosis symptoms of the retinoic acid-induced rats to a certain extent (Figure 9D–F).

##### H&E Staining Analysis

**Figure 10:**
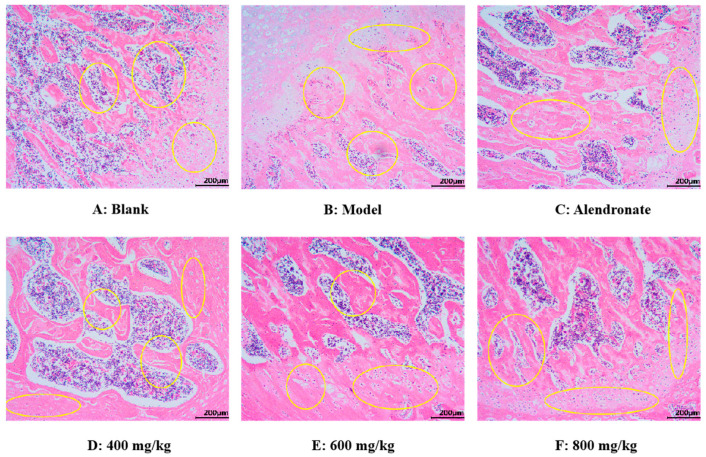
Morphological images of cross-section of femoral epiphysis using H&E staining method. Yellow circles indicate bone trabeculae and epiphyses.

As seen in Figure 10, using an optical electron microscope, the H&E staining images of the femoral epiphysis’ transverse section were captured. In the blank group (Figure 10A), the trabecular structure of the femur was normal and maintained well. In addition, the trabeculae have good continuity, close gaps, and uniform arrangement. Compared with the normal group, the trabecular structure of the femur in the model group was damaged, which was mainly manifested in the fracture of joints, thinning of thickness, reduction in number, sparse arrangement of trabeculae, wider gap, and no trabeculae in some areas (Figure 10B). These results indicated that retinoic acid can significantly damage the bone microstructure of rats and lead to osteoporosis. Compared with the model group, the TMP-Ca chelates could significantly restore the number of femoral trabeculae, increase trabecular thickness, and narrow trabecular space in rats (Figure 10D–F). Furthermore, compared to the low-dose group, the bone morphology in the TMP-Ca chelate medium- and high-dose groups was noticeably superior. These results indicate that the TMP-Ca chelates can effectively improve the structural damage of bone trabeculae and bone mass loss stimulated by retinoic acid, and enhance the microstructure of the bone.

### 3.3. Effect of TMP-Ca Chelates on the Protein Expression of OPG/TRAF6 Signaling Pathway

**Figure 11:**
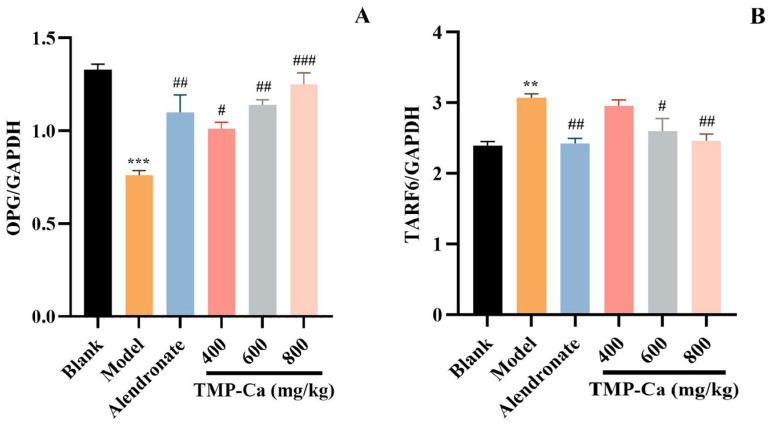
Effect of TMP-Ca chelates on the protein expression of the OPG/TRAF6 signaling pathway. (**A**) The protein expression of OPG; (**B**) the protein expression of TRAF6. All the values are means ± SD (*n* = 8). ^***^ *p* < 0.001 and ^**^ *p* < 0.01 vs. blank group; ^#^ *p* < 0.05, ^##^ *p* < 0.01, and ^###^ *p* < 0.001 vs. model group.

Figure 11 depicts the results of the protein expression of OPG/TRAF6 in the tibia of the retinoic acid-induced rats. In Figure 11A, the content of OPG protein in the tibia of retinoic acid-induced osteoporosis rats was 0.76 ± 0.03 (*p* < 0.001), which was notably less than that of rats in the blank group (1.33 ± 0.03). Moreover, TMP-Ca chelate treatment could increase the content of OPG protein, and the content was significantly increased to 1.25 ± 0.06 (*p* < 0.001) in the high-dose group of TMP-Ca chelates. In Figure 11B, the TRAF6 protein content within the model group was 3.07 ± 0.06 (*p* < 0.01), which was noticeably more than that in the blank group (2.39 ± 0.05). After the high-dose TMP-Ca chelate treatment, the TRAF6 protein content in the tibia of the retinoic acid-induced osteoporosis rats was significantly reduced to 2.46 ± 0.09 (*p* < 0.01). When OPG protein content increases, RANKL ligands are relatively reduced, which can reduce osteoclast differentiation, thereby inhibiting bone resorption and promoting bone formation.

## 4. Discussion

It has been demonstrated that fish, shrimp, and algae from seafood, as well as their processing by-products such as skin, scales, bones, and minced meat, or certain some low-value seafood rich in protein are ideal raw materials for the manufacture of Ca–peptide chelates [11,53]. Chen et al. prepared and determined the Ca chelation rate of the protein hydrolysates of *Auxis thazard* using papain and flavored enzymes, and purified and identified a Ca-chelating peptide of EPAH from the hydrolysate using SEC, RP-HPLC, and MS method [54,55]. In addition, the Ca-chelating peptides of GPAGPHGPPG [56] and KGDPGLSSPGK [57], and VLGYIQIR [58] were identified from the hydrolysates of Alaskan pollock skin collagen, Pacific cod, and Antarctic krill proteins.

By considering the unique protein structures derived from the diverse sources of blue foods and the specific cleavage sites of various proteases, our objective is to produce hydrolysis products that demonstrate optimal Ca chelating capacity. It is necessary to carefully analyze the amino acid sequences of the corresponding proteins, and optimize the protease and its enzymatic hydrolysis process with Ca-chelating ability as the screening criteria [19]. Trypsin is thought to be the most widely utilized commercial protease when it comes to Ca-chelating peptides made from blue food proteins [11]. For example, Ca-chelating peptides were prepared by hydrolyzing Antarctic krill, sea lamprey (*Muraenesox cinereus*) bone, and Alaskan pollock skin using trypsin. In addition, there are numerous combinations of enzymes, including trypsin, pepsin, and flavoring enzymes for the hydrolysis of tilapia scales, and trypsin and neutral proteases for the hydrolysis of Alaskan cod bones [56,57,58]. Therefore, in this experiment, the Ca chelation rate was used as the evaluation index to systematically compare the hydrolytic effect of four proteases on tuna meat protein (Figure 1A). The results showed that the protein hydrolysate prepared by trypsin had the highest Ca chelation ability. Therefore, trypsin was selected for the enzymolysis of tuna fish protein.

In order to optimize the enzymolysis process of trypsin, we designed a series of single-factor experiments to investigate the specific effects of enzymolysis dose, enzymolysis time, and solid–liquid ratio on the Ca chelation rate of tuna meat hydrolysate. Through the analysis of the experimental data, the optimum enzymolysis conditions were determined as follows: an enzyme dose of 3%, enzyme digestion time of 4 h, and material–liquid ratio of 1:10 (Figure 1B–D). On this basis, we further investigated the Ca chelation behavior of tuna meat hydrolysate under different environmental factors, including critical parameters such as pH, material–Ca ratio, chelation temperature, and chelation time. After the experimental comparison, we found that the Ca-chelating rate reached the most significant level when the material–Ca ratio was 1:10, the pH was 8.0, the chelation time was 50 min, and the temperature was at 50 °C (Figure 2).

**Figure 12:**
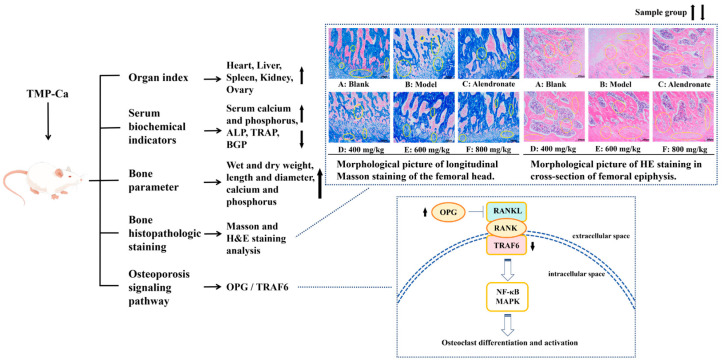
Mechanism of TMP-Ca chelates to ameliorate retinoic acid-induced osteoporosis of rats. The black arrow in the figure indicates the rising or falling trend of the sample group.

Peptide–Ca complexes have been shown in numerous studies to improve Ca absorption via diverse cellular pathways, increase the solubility of Ca in the gut, encourage precursor cell development, and improve the skeletal state of rats [59,60,61]. Therefore, a systematic experimental study was conducted using a retinoic acid-induced rat osteoporosis model to evaluate the potential efficacy of tuna meat peptide–Ca chelates (TMP-Ca) in osteoporosis prevention. As shown in Figure 12, through the analysis of various organ indexes in the retinoic acid-induced rat osteoporosis model, we found that the TMP-Ca chelates could significantly improve the decline of ovarian indexes caused by retinoic acid. Further serological tests showed that the TMP-Ca chelate treatment significantly increased serum Ca and P levels, and decreased the serum levels of key bone turnover markers such as ALP, TRAP, and BGP in the osteoporotic rats. In terms of bone tissue parameters, compared with the model group, the wet bone weight, dry bone weight, and Ca and *p* contents of the low-, medium- and high-dose groups of the TMP-Ca chelates were significantly improved. In addition, we further confirmed that different doses of the TMP-Ca chelates could effectively regulate the protein expression levels of OPG/TRAF6 in the retinoic acid-induced rat osteoporosis model.

In summary, the present study not only revealed the vital role of TMP-Ca chelates in preventing Ca loss and enhancing Ca absorption, but also confirmed its potential efficacy in preventing and improving osteoporosis. These findings provide a solid theoretical basis for applying tuna meat proteins in developing food-borne peptide–Ca supplementation functional products and offer new ideas and directions for research and development work in related fields.

## 5. Conclusions

This study comprehensively explored the efficient extraction of peptides from tuna meat and their chelation with CaCl_2_ to form peptide–calcium chelates. Additionally, it delved into the mechanism by which this novel product improves osteoporosis in animal models. Through a designed single-factor experiment, we optimized the production process for peptide–Ca chelates from tuna meat, determining the optimal enzymatic hydrolysis conditions (3% enzyme dosage, 4 h enzyme digestion time, and 1:10 material–liquid ratio) and Ca chelation conditions (1:10 material to Ca ratio, pH 8.0, 50 min duration, and 50 °C temperature). This achieved a high Ca chelation efficiency of up to 51.27 ± 1.42%. These research findings fill a gap in this field and provide valuable preparation schemes for similar products.

Furthermore, we systematically evaluated the intervention effects and mechanisms of action of TMP-Ca chelate agents on osteoporosis using a retinoic acid-induced rat model. The experimental results showed that the TMP-Ca chelates significantly improved the ovary index and alleviated organ damage while regulating serum-related indicators. Specifically, the TMP-Ca chelates increased the rats’ absorption of Ca and P while reducing serum ALP, TRAP, and BGP levels. These results indicate that the TMP-Ca chelates regulate osteoclasts and osteoblasts in the osteoporotic rats to promote bone resorption and formation as well as alleviate osteoporosis. For bone tissue parameters, such as femur and tibia dry weight and wet weight, length and diameter measurements, and changes in Ca and P content, our research demonstrated that the TMP-Ca chelates effectively mitigate bone loss caused by retinoic acid while improving bone quality and promoting skeletal development in the osteoporotic rats. A histological staining analysis visually displayed positive changes in the microscopic bone structure further confirming the protective effect of the TMP-Ca chelates on bones. Importantly, this study revealed that the TMP-Ca chelates regulate the OPG/TRAF6 signaling pathways at a molecular level to adjust bone metabolism providing new perspectives and strategies for treating osteoporosis. These findings not only deepen our understanding of the potential of Ca–peptide chelates from tuna meat in preventing osteoporosis, but also lay a solid scientific foundation for the deep development and high-value utilization of tuna resources.

## Figures and Tables

**Table 1:** Effect of TMP-Ca chelates on the indexes of major viscera in retinoic acid-induced osteoporosis rats.

Group	Heart (%)	Liver (%)	Spleen (%)	Kidney (%)	Ovary (%)
Blank	0.34 ± 0.06	2.53 ± 0.18 ^ab^	0.20 ± 0.08 ^ab^	0.55 ± 0.04 ^b^	0.16 ± 0.23 ^a^
Model	0.46 ± 0.01	2.92 ± 0.36 ^ab^	0.35 ± 0.01 ^a^	0.65 ± 0.18 ^ab^	0.06 ± 0.32 ^a^
Alendronate	0.36 ± 0.13	2.79 ± 0.58 ^ab^	0.15 ± 0.34 ^bc^	0.55 ± 0.62 ^ab^	0.11 ± 0.63 ^a^
TMP-Ca (400 mg/kg)	0.43 ± 0.03	3.24 ± 0.25 ^a^	0.27 ± 0.29 ^abc^	0.71 ± 0.78 ^a^	0.06 ± 0.93 ^b^
TMP-Ca (600 mg/kg)	0.39 ± 0.83	2.65 ± 0.42 ^b^	0.23 ± 0.62 ^c^	0.67 ± 0.34 ^ab^	0.10 ± 0.72 ^a^
TMP-Ca (800 mg/kg)	0.34 ± 0.06	2.55 ± 0.71 ^ab^	0.21 ± 0.03 ^ab^	0.55 ± 0.01 ^ab^	0.16 ± 0.91 ^ab^

All the values are means ± SD (*n* = 8). ^a–c^ No significant difference between the same letters (*p* > 0.05).

## Data Availability

The original contributions presented in the study are included in the article, further inquiries can be directed to the corresponding authors.
